# Combined toxicity of cyanotoxins and anthropogenic contaminants on *Chlorella vulgaris*: insights from single and binary mixture exposures

**DOI:** 10.1007/s10646-026-03171-z

**Published:** 2026-10-23

**Authors:** Carlos Pinheiro, Joana Azevedo, Alexandre Campos, Patrícia V. Silva, Vítor Vasconcelos, Susana Loureiro

**Affiliations:** 1 CESAM-Centre for Environmental and Marine Studies & Department of Biology, Universidade de Aveiro, Aveiro, 3810-193 Portugal; 2 CIIMAR, Interdisciplinary Centre of Marine and Environmental Research of the University of Porto, Terminal de Cruzeiros do Porto de Leixões, Av. General Norton de Matos, s/n, Matosinhos, 4450-208 Portugal; 3 Department of Biology, Faculty of Sciences, University of Porto, Rua do Campo Alegre, Porto, 4169-007 Portugal

**Keywords:** Microcystin-LR, Cylindrospermopsin, Terbuthylazine, Cadmium, Microalgae, Synergism

## Abstract

Aquatic organisms in eutrophic brackish and freshwater systems are chronically exposed to natural and anthropogenic contaminants, including cyanotoxins, metals and pesticides, mainly driven by cyanobacterial blooms and human activities. However, the environmental risks posed by their combinations to the aquatic environment remain poorly understood. This study investigated the growth response of *Chlorella vulgaris* exposed to four single-chemical compounds (microcystin-LR, cylindrospermopsin, terbuthylazine, and cadmium) and five binary mixtures for 7 days. In single-chemical exposures, terbuthylazine was the most toxic to *C. vulgaris*, followed by cadmium, cylindrospermopsin and microcystin-LR. The obtained growth data were compared with concentration addition predictions based on individual toxicities using the MIXTOX tool, testing deviations for synergistic/antagonistic interactions, dose ratio and dose level dependencies. Mixture effects varied with composition and exposure time. Antagonism was the prevailing type of interaction between the chemical compounds after 4 days of exposure, while dose level dependency was the only deviation observed after 7 days. Regarding dose level dependency, synergism was observed at low doses of each chemical in three of the five tested binary mixtures. Time was an important factor in the change in the toxicity pattern. This synergistic effect poses a significant risk to aquatic organisms exposed simultaneously to cyanotoxins and anthropogenic contaminants in the environment.

## Introduction

The global intensification of agricultural and industrial activities has increased the extent of eutrophication in brackish and freshwater ecosystems, leading to a worldwide increase in harmful cyanobacterial blooms (HABs) (Huisman et al. 2018). Climate change, especially the predicted rise in temperatures and altered precipitation patterns, is expected to further enhance HAB frequency, intensity, persistence, and geographic distribution (Heil and Muni-Morgan 2021; Chorus and Welker 2021). Such blooms are a major ecological and human health concern because many bloom-forming cyanobacterial species can produce potent cyanotoxins that can also accumulate in the food chain (Igwaran et al. 2024). Among these, the hepatotoxic microcystins (MCs) and the cytotoxin cylindrospermopsin (CYN) are the most frequently detected in HABs in brackish and freshwater environments (Codd et al. 2005; van Apeldoorn et al. 2007; Dreher et al. 2022).

During HAB development, MCs are mostly retained within the cyanobacterial cells, whereas CYN is continuously released into the water due to its hydrophilic properties and apparent membrane permeability (Shaw et al. 1999; Wiegand and Pflugmacher 2005). Cell senescence and/or lysis can result in high environmental concentrations of both cyanotoxins. MCs levels may exceed the World Health Organization guideline values of 1 µg L^− 1^ in drinking water and 10 µg L^− 1^ in recreational waters. Although MC levels in surface waters are generally < 100 µg L^− 1^, they can exceed 1000 µg L^− 1^ under certain environmental conditions, such as pH, temperature, and nutrient levels (Melaram et al. 2022). MC concentrations ranging from 4 to 6500 µg L^− 1^ were detected in surface waters used for irrigation in several countries, including Finland, Turkey, India, and Morocco (Machado et al. 2017). Concentrations of CYN in water can reach values up to 126 µg L^− 1^ (Machado et al. 2017). These high levels, which may become more common as the prevalence and magnitude of HABs increase (Paerl and Huisman 2009; Paerl and Paul 2012), prompted this study to examine a range of concentrations, including extremely high levels. As MCs and CYN are highly stable and persist for days under conditions found in most natural water bodies (Lahti et al. 1997; Wormer et al. 2008; Zhang et al. 2022; Dang et al. 2023; Pawlik-Skowrońska et al. 2023), a range of aquatic organisms, including microalgae, can be exposed and potentially affected by cyanotoxins (Diez-Quijada et al. 2021; Teneva et al. 2023).

MCs and CYN may disrupt important cellular processes, such as growth of microalgae and fish (B-Béres et al. 2012; Casas-Rodriguez et al. 2022), photosynthesis and detoxification pathways in aquatic plants and green algae (Pietsch et al. 2001; Wiegand et al. 2002; Mohamed 2008; Teneva et al. 2023) and reproduction in aquatic snails and fish (Kinnear et al. 2007; Casas-Rodriguez et al. 2022). MCs inhibit protein phosphatases 1 and 2 A in mammals and higher plants (Mackintosh et al. 1990; Teneva et al. 2023). Moreover, numerous studies have demonstrated that oxidative stress may play a significant role in the toxicity mechanism of MCs in aquatic animals, plants, and algae (Pflugmacher 2004; Mohamed 2008; Amado and Monserrat 2010; Bártová et al. 2011; Teneva et al. 2023). In plants, MCs also induce histone H3 hyperphosphorylation, leading to incomplete chromatid segregation and the formation of micronuclei (Máthé et al. 2013). CYN inhibits glutathione and protein synthesis (Runnegar et al. 1995; Froscio et al. 2008; Adamski and Kaminski 2021) and potentially interferes with DNA structure in mammals (Shaw et al. 2000). Cytochrome P450-generated metabolites are likely to mediate its toxic mechanism (Humpage et al. 2005). In plants, CYN has been recognised by alterations in microtubule formation (including preprophase bands) and spindle-phragmoplast disruption, which are directly related to protein synthesis inhibition (Metcalf et al. 2004; Máthé et al. 2013; Ziesemer et al. 2022).

Concomitant with eutrophication and cyanobacterial proliferation, agriculture and industry have increased the release of high amounts of persistent anthropogenic contaminants into aquatic systems, such as metals and pesticides, which can be toxic to aquatic organisms, including microalgae (Bišová et al. 2003; Weiner et al. 2007; Huang et al. 2009; Pérez et al. 2011; Akinnawo 2023).

Aquatic organisms may be simultaneously exposed to multiple natural and anthropogenic environmental contaminants from various sources, which can produce effects on aquatic communities that exceed those of individual compounds. While combined effects of cyanotoxins (especially MC-LR) and anthropogenic contaminants have been reported on invertebrates (Yang et al. 2011, 2012; Cao et al. 2014; Wan et al. 2021), fish (Notch et al. 2011; Sun et al. 2012; Wei et al. 2020), and plants (Ge et al. 2012; Wang et al. 2011, 2012, 2017; Wan et al. 2019) data for freshwater microalgae remain scarce (Hernández-García and Martínez-Jerónimo 2020).

The present study therefore assessed the growth response of the freshwater algae *Chlorella vulgaris* exposed to cyanotoxins and anthropogenic contaminants, both singly and in binary mixtures. Four chemical compounds were selected: (1) cyclic heptapeptide MC-LR as the most common and toxic variant of MCs (Dittmann and Wiegand 2006), (2) tricyclic alkaloid CYN due to its worldwide occurrence (Falconer and Humpage 2006; Quesada et al. 2006; Fastner et al. 2007; González-Blanco et al. 2020), (3) triazine-ring herbicide terbuthylazine (TBA), and (4) the non-essential metal cadmium (Cd). TBA was selected because it is frequently detected in surface waters (Palma et al. 2009; Pérez et al. 2010; Tasca et al. 2018), and acts at the photosystem II (PSII) of photoautotroph organisms, inhibiting the photosynthetic electron transport (Faust et al. 2001; Lettieri et al. 2022). Cd was selected because it is commonly found in the aquatic environment due to a variety of anthropogenic and natural sources, and is known to induce oxidative stress (Pinto et al. 2003; Leonard et al. 2004; Valko et al. 2005), and to compete with the essential Ca^2+^ site in PSII during photoactivation (Faller et al. 2005; Chu et al. 2018; Branca et al. 2020). We hypothesised that the growth responses of *C. vulgaris* would be adversely affected by the tested compounds, both individually and in binary mixtures. Given that these compounds have distinct mechanisms of action but may affect related physiological processes, we further hypothesised that their combined effects could deviate from those predicted based on single-compound exposures, resulting in either synergistic or antagonistic interactions.

## Materials and methods

### Test chemicals

Chemical substances used in this study were cadmium chloride anhydrous (CdCl_2_, 99% purity, FLUKA, Sigma-Aldrich), TBA (formula: C_9_H_16_ClN_5_, molecular weight: 229.7 Daltons) tested as a commercial formulation (SAPEC with 500 g active ingredient L^− 1^), purified MC-LR (formula: C_49_H_74_N_10_O_12_, molecular weight: 994 Daltons, 97% purity) and purified CYN (formula: C_15_H_21_N_5_O_7_S, molecular weight: 415 Daltons, 98.7% purity). The cyanotoxins were extracted and subsequently purified by a Water Alliance e2695 HPLC system (Milford, Massachusetts, USA) coupled with a PDA 2998. All TBA concentrations in the experiments are expressed as active ingredients per litre of Z8 medium (Kotai 1972).

Concentrations were measured by HPCL-PDA on two stock solution samples and on 32 and 29 exposure medium samples from single and combined exposures for MC-LR and CYN, respectively. The stability of MC-LR and CYN was also monitored throughout the exposure period using HPLC-PDA, under the same experimental conditions. Actual concentrations of Cd and TBA were not analytically determined because the experiments were conducted in 96-well microplates with very small test volumes, which severely limited the sample volume available for chemical analysis. Nominal concentrations were considered appropriate for both Cd and TBA, as Cd is an inorganic element that does not degrade during the exposure period, whereas TBA has been reported to be stable in aqueous solution under comparable experimental conditions, with negligible hydrolysis reported (Lewis et al. 2016; Queirós et al. 2018a).

### Test organism and cyanobacterial strains

All experiments were conducted with the freshwater algae *C. vulgaris* (LEGE Z-001, acquired from the University of Coimbra). This species was chosen because it is a widespread freshwater primary producer that co-occurs with cyanobacterial blooms and contaminants, and is a well-established model organism. Axenic cultures of *C. vulgaris* were maintained in Z8 medium (Kotai 1972) and incubated at 25 ± 2 °C under a light intensity of 10 µmol m^− 2^.s^− 1^ photon irradiance with a 14 h light and 10 h dark photoperiod. Cultures were aerated with ambient air filtered through a 0.22 μm membrane to maintain sterile conditions. For the maintenance of lab cultures and the initiation of new ones, *C. vulgaris* was harvested while still in the exponential growth phase (7–8 days old) and inoculated into fresh Z8 medium at a cell density of 5 × 10^5^ cells mL^− 1^.

The MC-LR- and CYN-producing cyanobacterial strains used in this study were *Microcystis aeruginosa* (LEGE 91094) and *Aphanizomenon ovalisporum* (LEGE X-001, from the LEGE culture collection). The strain *M. aeruginosa* was isolated from Lagoa de Mira, Portugal, and has been reported to produce primarily the MC-LR variant, which accounts for approximately 95% of the total intracellular MCs (Pereira et al. 2009a). The strain *A. ovalisporum* was isolated from Lake Kinneret, Israel and verified by HPLC-PDA to produce only CYN (unpublished laboratory data). Species identification was confirmed in the laboratory through morphological analysis. Cultures of both cyanobacterial species were maintained in Z8 medium under the same conditions as for *C. vulgaris* cultures, as described above. After 3–4 weeks of growth, cyanobacterial biomass was collected by centrifugation (*M. aeruginosa*) or filtration (*A. ovalisporum*), then frozen at -80 °C and lyophilised (Pinheiro et al. 2013). Optical microscopy confirmed that the cultures used were unialgal (Pinheiro et al. 2013).

### MC-LR extraction, purification and quantification

MC-LR was extracted from lyophilised *M. aeruginosa* biomass using the method described by Pinheiro et al. (2013). Briefly, the freeze-dried cells were extracted with MeOH 75% (v/v) under continuous stirring for 20 min at room temperature. The sample was then sonicated in a bath for 15 min at room temperature and ultrasonicated on ice at 60 Hz (VibraCell 50-sonics & Material Inc. Danbury, CT, USA), with 5 cycles of 1 min. The homogenate was then centrifuged (10,000 g, 15 min) to remove cell debris, and the supernatant was collected and applied to solid-phase extraction. The toxin MC-LR was eluted using MeOH 80% (v/v) and concentrated by rotary evaporation at 35 °C (Pinheiro et al. 2013).

MC-LR was thereafter purified and quantified by HPLC-PDA. A reversed-phase column (Phenomenex Luna RP-18, 25 cm × 10 mm, 10 μm) kept at 35 °C was used for MC-LR purification. The gradient elution used was a mixture of MeOH and water, acidified with 0.1% trifluoroacetic acid (TFA) at a flow rate of 2.5 mL min^− 1^. The injected volume was 500 µL. Peak purity and percentage of purified MC-LR were calculated at 214 and 238 nm. The MC-LR fraction was then evaporated under a stream of nitrogen, and the residue was resuspended in the culture medium to the desired concentration. For MC-LR quantification, a reversed-phase column (Merck Lichrospher RP-18 endcapped, 25 cm × 4.6 mm, 5 μm) equipped with a guard column (4 × 4 mm, 5 μm), both kept at 45 °C, was used. The gradient elution consisted of (A) MeOH + 0.1% TFA and (B) H_2_O + 0.1% TFA (55% A and 45% B at 0 min, 65% A and 35% B at 5 min, 80% A and 20% B at 10 min, 100% A at 15 min, 55% A and 45% B at 15.1 and 20 min) with a flow rate of 0.9 mL min^− 1^. The injected volume was 20 µL. The PDA range was 210–440 nm, with a fixed wavelength at 238 nm. The MC-LR was identified by comparing its spectra and retention time with those of a standard MC-LR (≥ 95% purity, Sigma-Aldrich). The system was calibrated using a set of seven dilutions of MC-LR standard (0.5–20 µg mL^− 1^) in MeOH 50%. The limit of detection of the MC-LR in the HPLC-PDA system is 0.2 µg mL^− 1^.

After analysis, the final concentration of the MC-LR stock solution was 673.3 mg L^− 1^, and its purity was 97% according to HPLC chromatograms. Purified MC-LR was then diluted in Z8 medium to the concentration range used in experiments.

### CYN extraction, purification and quantification

CYN was extracted from lyophilised *A. ovalisporum* biomass according to the method described by Pinheiro et al. (2013). Briefly, the freeze-dried cells were extracted with distilled H_2_O + TFA 0.1% (v/v) by continuous stirring for 1 h at room temperature. The sample was then sonicated in a bath for 15 min and ultrasonicated on ice at 60 Hz for 5 cycles of 1 min. After the extraction step, the homogenate was centrifuged (20,000 g, 20 min), and the supernatant was collected and stored at -20 °C.

The same HPLC system was used to purify and quantify the CYN content in the extract. A reversed-phase column (Phenomenex Gemini C18 column, 250 × 10 mm, 5 μm) maintained at 40 °C was used for CYN purification. Isocratic elution was used with 5% (v/v) MeOH containing 2 mM sodium 1-heptanesulfonate monohydrate, at a flow rate of 3 mL min^− 1^. The injection volume was 500 µL. Peak purity and percentage of purified CYN were calculated at 262 nm. The CYN fraction was then evaporated by speed-vac at 30 °C and redissolved in the culture medium to the desired concentration. A reversed-phase column (Waters Atlantis^®^ HILLIC, 250 × 10 mm, 5 μm) was kept at 40 °C for CYN quantification. The isocratic elution was the same as that used for the purification step, but with a flow rate of 0.9 mL min^− 1^ and an injected volume of 10 µL. The PDA range was 210–400 nm with a fixed wavelength of 262 nm. The CYN was identified by comparing its spectra and retention time with those of a standard CYN (100% purity, Cork University, Ireland). The system was calibrated using a set of seven dilutions of CYN standard (0.5–20 µg mL^− 1^) in ultrapure water. The limit of detection for CYN in the HPLC-PDA system is 0.3 µg mL^− 1^.

After analysis, the final concentration of the CYN stock solution was 608.7 mg L^− 1^, and its purity exceeded 98%, as indicated by HPLC chromatograms. Purified CYN was diluted in Z8 medium to the concentration range used in experiments.

### Experimental design

#### Single-chemical exposures

The growth inhibition tests with the freshwater algae *C. vulgaris* were performed in 96-well polystyrene microplates, as an adaptation of the OECD 201 (OECD 2011) based on the method described by Gantar et al. (2008), to reduce test volumes and the amount of cyanotoxins required for the selected concentration range. The log-phase-growing microalgae at a cell concentration of approximately 5 × 10^5^ cells mL^− 1^ were exposed to the chemical compounds for 7 days, and algal growth was determined according to the OECD 201 Guideline (2011). Nine concentrations of Cd and TBA, plus a negative control (Z8 medium containing *C. vulgaris* only) were tested with 5 replicates each. Nominal concentrations ranged from 0.25 to 64 mg L^− 1^ for Cd and from 0.001 to 0.5 mg L^− 1^ for TBA. Five concentrations of MC-LR (1.2 to 37.3 mg L^− 1^) and CYN (0.4 to 16.7 mg L^− 1^) were selected based on Pinheiro et al. (2013). These high concentrations were required to obtain reliable EC_50_ estimates, as lower concentrations caused little to no growth inhibition in *C. vulgaris* (Pinheiro et al. 2013). Three replicates (wells) per treatment were used, each containing 200 µL of test solution (with or without the chemical substance) prepared in fresh culture medium. The lower number of replicates for the cyanotoxins was due to the limited availability of purified MC-LR and CYN extracted from cyanobacterial biomass.

Microplates were sealed with perforated parafilm (to reduce evaporation and allow gas exchange) and incubated for 7 days under the same temperature and illumination conditions as for the microalgal cultures. Algal growth was measured indirectly by determining the optical density at 750 nm using a microplate reader (PowerWave, BioTek, Vermont, USA). Readings were taken at the beginning of each experiment, after 4 and extended to 7 days of exposure to assess time-dependent changes in the toxicity of the individual compounds and their mixture.

Before measuring optical density (on days 4 and 7), the well content was resuspended with a pipette. All the optical density values were then converted into cells ml^− 1^ using the equation: C = 3.00 × 10^7^ × Abs + 2.17 × 10^5^ (R^2^ = 0.99), where C is the algae concentration (cells ml^− 1^), and Abs is the absorbance obtained at 750 nm. The average specific growth rate was calculated as described by Pinheiro et al. (2013). The pH values were recorded at the beginning and end of the experiments. To verify test validity, the reference substance, potassium dichromate, was used at three concentrations (5, 10, and 20 mg L^− 1^), with 5 replicates each.

#### Binary mixture exposures

The growth inhibition tests with binary mixtures were conducted using the same methodology as in the single-chemical tests. The following binary combinations were tested: MC-LR and TBA; MC-LR and Cd; CYN and TBA; CYN and Cd; and TBA and Cd. A partial factorial experimental design was used for the MC-LR- and CYN-containing mixtures because the highest cyanotoxin concentrations, included in the single-exposure assays to obtain reliable EC_50_ estimations, were not tested in the mixture experiments. Consequently, not all combinations of concentrations were tested. In contrast, all concentration combinations were evaluated for the TBA and Cd mixture, enabling a full factorial design.

**Fig. 1:**
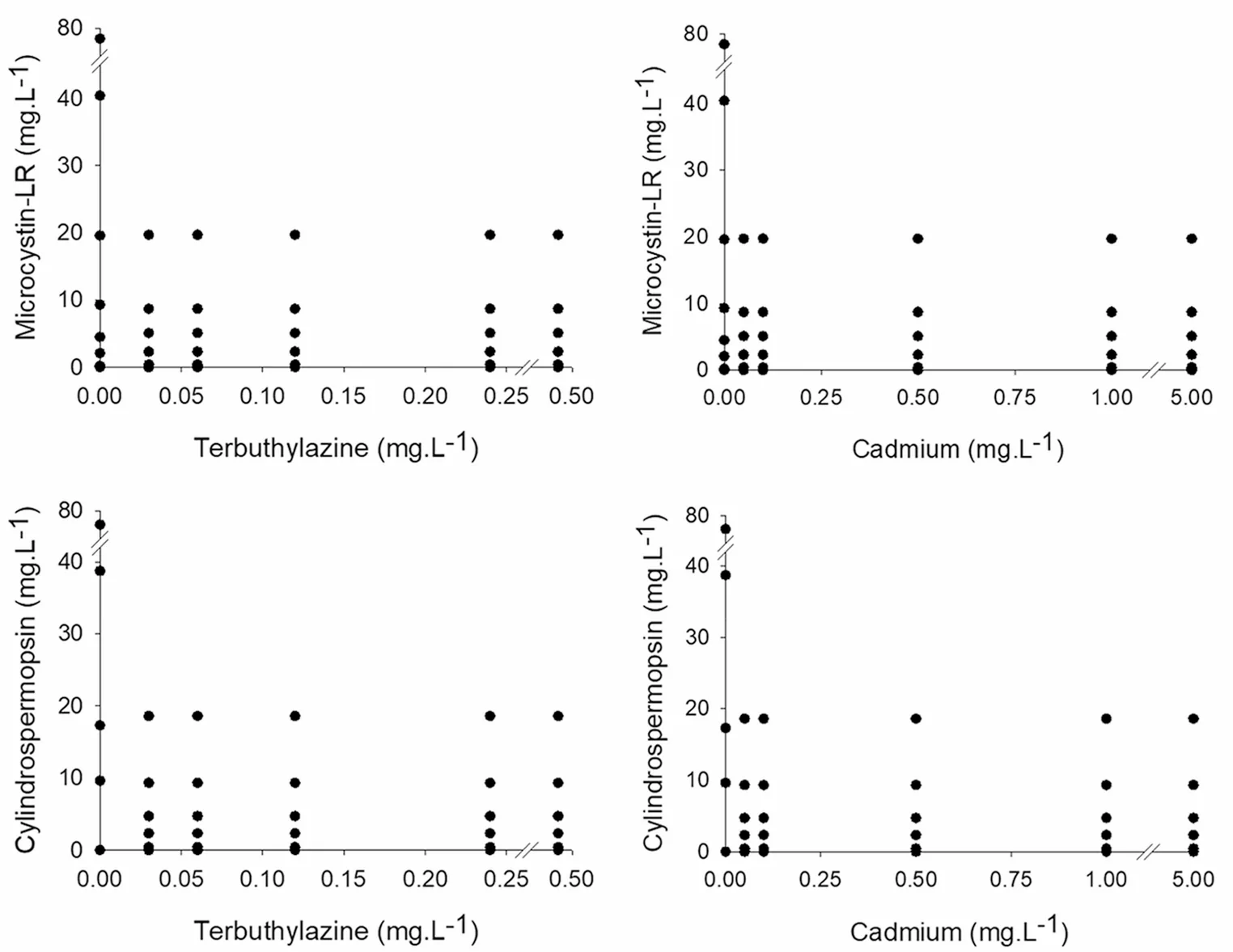
A schematic ray design of the combinations used for the microcystin-LR and terbuthylazine, microcystin-LR and cadmium, cylindrospermopsin and terbuthylazine, cylindrospermopsin and cadmium mixtures

For the MC-LR combination with TBA and Cd, the experimental design consisted of single exposures to 7 concentrations of MC-LR and 5 concentrations of TBA or Cd and 25 combinations of MC-LR and TBA or MC-LR and Cd (Fig. 1). The nominal concentrations for MC-LR single exposures ranged from 0.5 to 80 mg L^− 1^ and for combined exposures, from 0.5 to 20 mg L^− 1^. Such high concentrations were selected for MC-LR single exposures to calculate EC_50_ values, as toxicity predictions for binary mixture exposures are based on full dose-response curves and, in previous work, effects at lower concentrations did not cause 50% inhibition of *C. vulgaris* growth rates (Pinheiro et al. 2013).

For the CYN combination with TBA and Cd, the experimental design consisted of single exposures to 4 concentrations of CYN and 5 concentrations of TBA and Cd and 25 combinations of substances for each case (Fig. 1). The nominal concentrations for CYN single exposures ranged from 10 to 80 mg L^− 1^ and for combined exposures, from 0.5 to 20 mg L^− 1^. Again, a high concentration range of CYN was used in single exposures to obtain reliable EC_50_ estimates, based on previous work showing that lower concentrations caused only weak growth inhibition (< 10%) in *C. vulgaris* (Pinheiro et al. 2013).

**Fig. 2:**
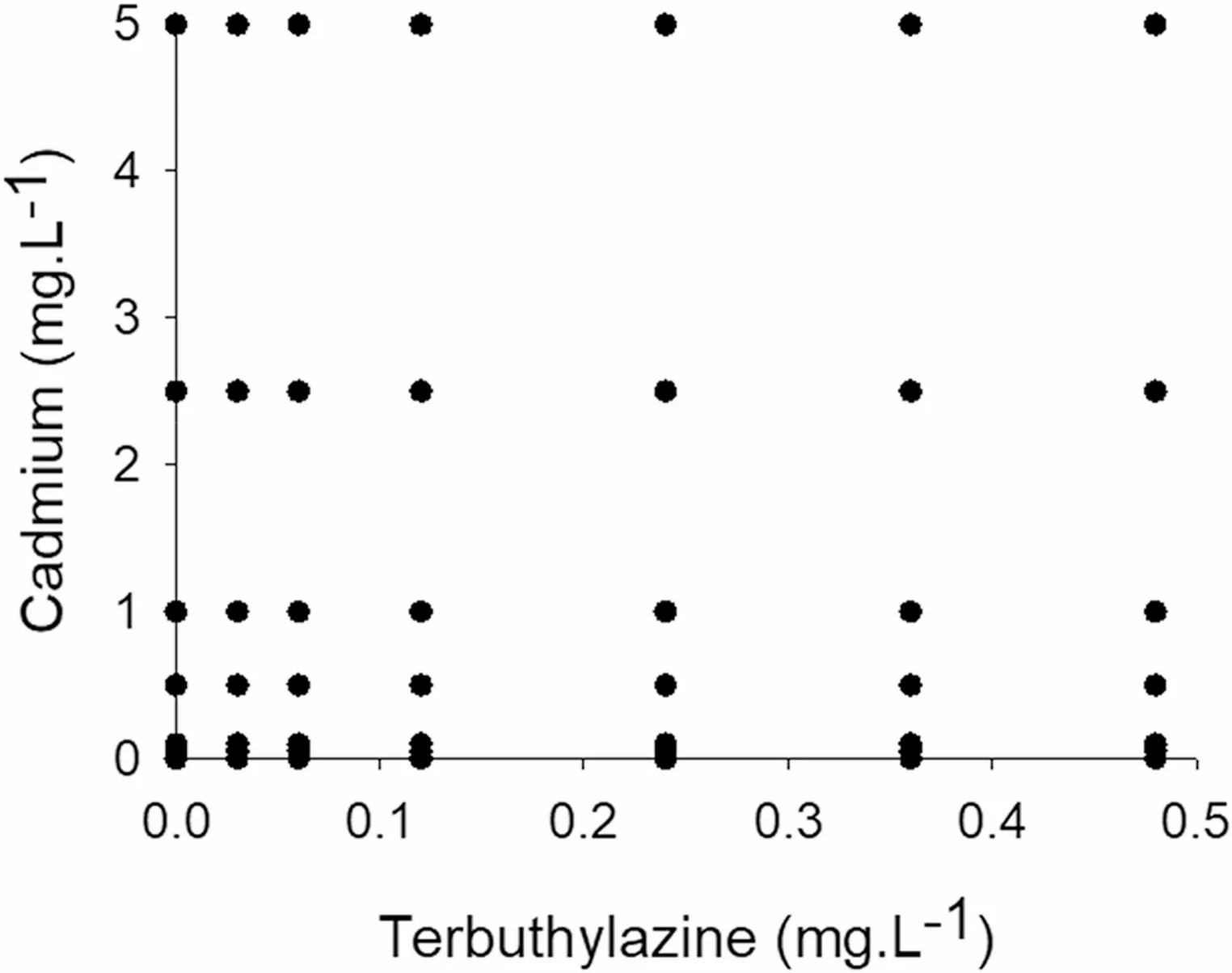
A schematic full factorial design of the combinations used for the terbuthylazine and cadmium mixture

For the TBA and Cd combination, the experimental design consisted of single exposures to six concentrations of each chemical and 36 combinations of both chemicals (Fig. 2). The nominal concentrations ranged from 0.03 to 0.48 mg L^−^¹ for TBA and from 0.05 to 5 mg L^−^¹ for Cd, both in single and combined exposures.

In the binary mixture experiments, individual and mixture exposures were conducted simultaneously to control for variations in organism sensitivity between experiments and to ensure direct comparison with the mixture responses. Three replicates were used for both the concurrent single- and mixture experiments, to ensure direct comparison under identical experimental conditions.

### Data analysis

The no observed effect concentrations (NOEC) and the lowest observed effect concentrations (LOEC) were determined by a one-way ANOVA (SigmaPlot software), followed by Dunnett’s multiple comparison test to detect differences between data. When the normality test and data transformation procedures failed, a non-parametric Kruskal-Wallis ANOVA on ranks was used with multiple comparisons Dunn’s method. All significant differences were established at *p* ≤ 0.05.

When possible, EC_50_ and slope values for each chemical compound in single-exposure conditions were calculated using the three-parameter logistic equation. When it was not possible to fit this curve to obtain reliable EC_50_ estimates, the analysis in the MIXTOX tool was conducted with fixed equivalent concentration transformation (ECT) and slope parameters, as described by Loureiro et al. (2010).

The observed effects were compared with the expected mixture effects calculated from single-chemical exposures using the concentration-addition (CA) model described by Loewe and Muischnek (1926). The conceptual model for CA, which assumes that the components in the mixture have similar modes of action, was chosen to fit the binary mixture data. This model was selected because it is considered more conservative than the independent action model, which assumes dissimilar modes of action among mixture components, and therefore regarded as a precautionary default assumption (EFSA 2015). Moreover, the CA model allows prediction of mixture toxicity where one (or both) of the stressors does not exert a complete concentration-response curve or a significant effect (Backhaus et al. 2004; Boedeker et al. 1993). However, the toxic behaviour of a mixture may deviate from this conceptual model, thereby being more or less severe than expected. Deviations from the CA model, such as synergism/antagonism (S/A), dose ratio (DR) dependency and dose level (DL) dependency, were obtained by the addition of two parameters (a and b) to the mathematical model that describes CA [see in more detail in Jonker et al. (2005)]. The biological interpretation of these additional deviation parameters is summarised in Table 1. The tool used to analyse and compare the data in the present study was MIXTOX, which allowed fitting the CA model to the data and extending it to test for deviations. The data fitted to the CA model, or their deviations, were compared using the method of maximum likelihood, and the best fit was chosen based on the Chi-square test at a significance level of 0.05. When a deviation from the CA model was observed, the pattern of effects was deduced directly from the parameter values listed in Table 1.

**Table 1:** Interpretation of additional parameters (*a* and *b*) that define the functional form of deviation patterns from concentration addition

Deviation pattern	Concentration addition	
	Parameter *a*	Parameter *b*
Synergism/Antagonism (S/A)	***a*** **> 0**: antagonism	
	***a*** **< 0**: synergism	
Dose ratio-dependent (DR)	***a*** **> 0**: antagonism except for those mixture ratios where the negative b value indicates synergism	***b***_***i***_**> 0**: antagonism where the toxicity of the mixture is caused mainly by toxicant *i*
	***a*** **< 0**: synergism except for those mixture ratios where positive b value indicates antagonism	***b***_***i***_**< 0**: synergism where the toxicity of the mixture is caused mainly by toxicant *i*
Dose level-dependent (DL)	***a*** **> 0**: antagonism low dose level and synergism high dose level	***b***_***DL***_**> 1**: change at lower EC_50_ level
		***b***_***DL***_**= 1**: change at EC_50_ level
	***a*** **< 0**: synergism low dose level and antagonism high dose level	**0 <** ***b***_***DL***_**< 1**: change at higher EC_50_ level
		***b***_***DL***_**< 0**: no change, but the magnitude of S/A is effect level dependent

EC_50_ is the median effect concentration

Adapted from Jonker et al. (2005)

Whenever it was not possible to observe a full dose-response curve for a particular component of a binary mixture, synergistic ratios (SR) were calculated to estimate the magnitude of the synergistic inhibition of growth rates exerted by that mixture. The EC_50_ values for the chemical in the mixture with a full dose-response curve were estimated in the absence and presence of each concentration of the second chemical, for which no full dose-response curve was obtained. This approach was carried out, when feasible, using the same three-parameter logistic curve (the dose-response curve used in the MIXTOX tool). The SRs were then calculated by dividing the EC_50_ value for the single exposure of the first chemical by the EC_50_ value for the same chemical when combined with each of the second chemical treatments. SRs of 1.0 indicate no change in the EC_50_ of the first chemical in the presence of the second chemical, whereas SR values > 1.0 and < 1.0 indicate a decrease and an increase in the EC_50_, respectively, relative to single exposure.

## Results

### Chemical analysis

In all single tests performed, pH did not differ significantly between treatments nor between the initial and final measurements, ranging from 7.3 to 7.6 and 9.1–9.5 for MC-LR and CYN, and between 7.8 and 8.2 and 8.6–9.5 for Cd and TBA. This indicates that this parameter did not interfere with *C. vulgaris*’s response to the chemical exposures.

MC-LR and CYN concentrations were determined by HPLC-PDA, with deviations exceeding 20% from nominal concentrations, therefore, all calculations were based on measured concentrations. Stability analyses using HPLC-PDA showed no degradation of MC-LR or CYN throughout the 7-day exposure period.

### Single chemical exposures

The NOEC, LOEC and EC_50_ values for the four single-chemical exposures are reported in Table 2, along with the data obtained in the single-chemical exposures from the binary mixture experiments. After 4 days of exposure, TBA was the most toxic to *C. vulgaris*, about 5 times more toxic than Cd, which, in turn, was approximately 28 times more toxic than CYN. After 7 days of exposure, the toxicity difference between TBA and Cd decreased, with TBA only 2.5 times more toxic. In contrast, the difference in toxicity between Cd and CYN was much greater, with Cd approximately 46 times more toxic than CYN. MC-LR showed the lowest toxicity in both exposure periods.

**Table 2:** NOEC, LOEC, and EC_50_ values (with corresponding standard errors), in mg L^− 1^, for the effect of selected chemical substances on the growth rate of *Chlorella vulgaris* exposed for 4 and 7 days in Z8 medium

Substances	NOEC		LOEC		EC_50_ (± SE)		Experiment
	Day 4	Day 7	Day 4	Day 7	Day 4	Day 7	
Microcystin-LR (MC-LR)	> 37.3	23.9	> 37.3	37.3	> 37.30	> 37.30	Single exposure experiments^a^
	40.4	40.4	78.9	78.9	> 78.9	> 78.9	Mixture experiment with TBA
	19.6	40.4	40.4	78.9	> 78.9	> 78.9	Mixture experiment with Cd
Cylindrospermopsin (CYN)	4.4	> 16.7	8.5	> 16.7	> 16.70 (n.d.)	> 16.70 (n.d.)	Single exposure experiments^a^
	17.3	9.6	38.7	17.3	49.21 (± 3.29)	47.67 (± 2.98)	Mixture experiment with TBA
	17.3	9.6	38.7	17.3	56.34 (± 2.73)	51.26 (± 1.72)	Mixture experiment with Cd
Terbuthylazine (TBA)	0.01	0.025	0.025	0.05	0.39 (± 0.03)	0.39 (± 0.02)	Single exposure experiments
	0.12	0.03	0.24	0.06	0.37 (± 0.03)	0.46 (± 0.03)	Mixture experiment with MC-LR
	0.12	0.06	0.24	0.12	> 0.48	> 0.48	Mixture experiment with CYN
	< 0.03	0.03	0.03	0.06	0.35 (± 0.01)	0.46 (± 0.02)	Mixture experiment with Cd
Cadmium (Cd)	1	< 0.25	2	0.25	1.17 (± 0.19)	0.88 (± 0.08)	Single exposure experiments
	0.1	0.1	0.5	0.5	0.92 (± 0.08)	0.76 (± 0.04)	Mixture experiment with MC-LR
	1	0,1	5	0.5	4.15 (n.d.)	1.80 (± 0.14)	Mixture experiment with CYN
	0.1	< 0.05	0.5	0.05	1.27 (± 0.08)	0.88 (± 0.03)	Mixture experiment with TBA

^a^Results for *C. vulgaris* from Pinheiro et al. (2013)

NOEC is the no observed effects concentration, LOEC is the lowest observed effect concentration, and EC_50_ is the median effect concentration

### Binary mixture exposures

In the mixture experiments, single exposures to TBA (in the binary mixture with CYN) and MC-LR did not achieve 50% growth inhibition in *C. vulgaris*, preventing valid EC_50_ calculations. Consequently, MIXTOX analyses were performed using estimated and fixed EC_50_ and slope parameters. The EC_50_ values across experiments were similar, indicating good methodological reproducibility (Table 2).

**Fig. 3:**
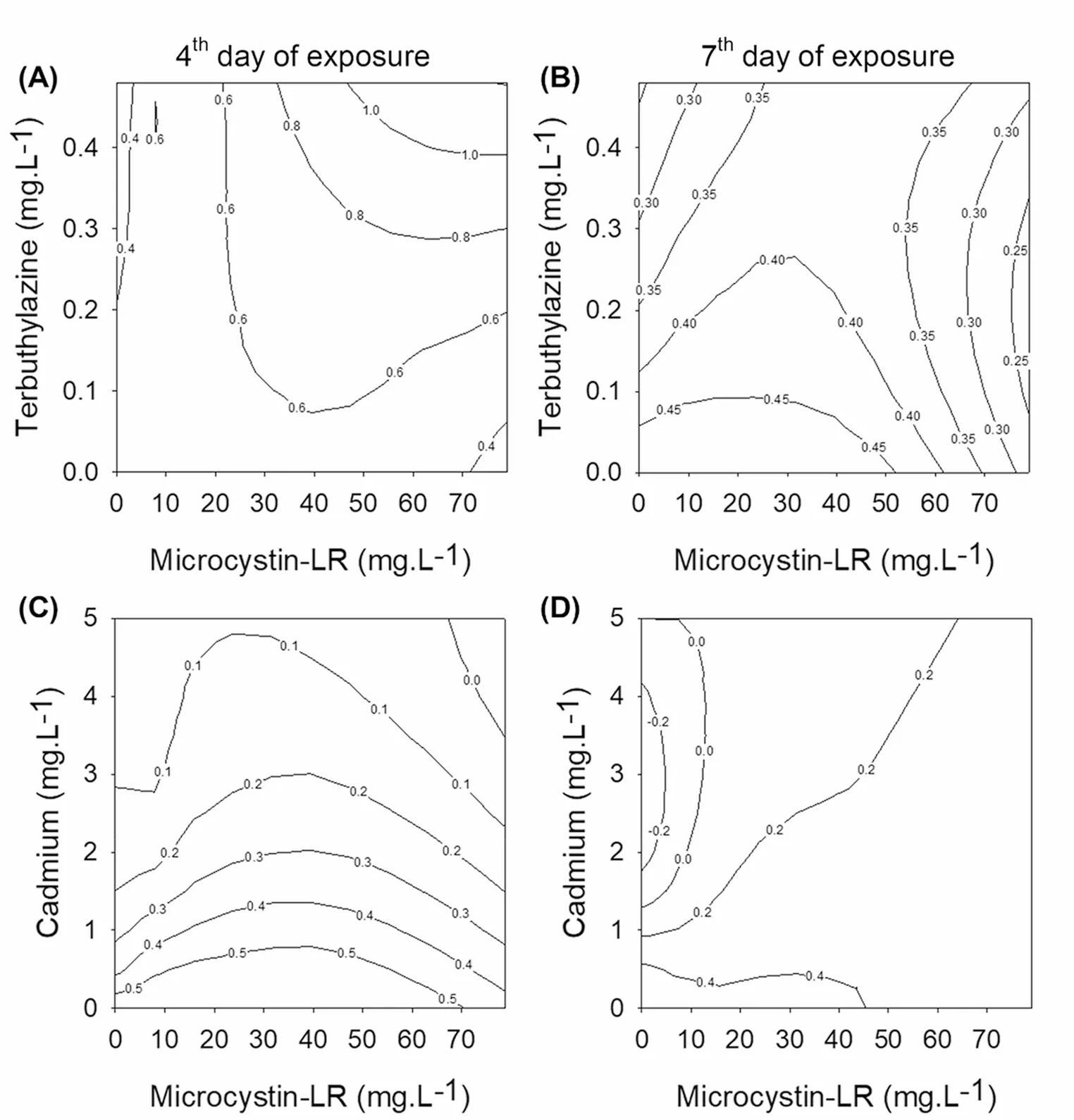
Concentration-response relationship for the binary mixtures of microcystin-LR and terbuthylazine and microcystin-LR and cadmium tested on the growth rate of *Chlorella vulgaris* after 4 and 7 days of exposure (2D isobolic surface). **A**,** B** and **D** show a dose level-dependent deviation from the CA model; **C** shows an antagonistic deviation from the CA model. Concentrations of microcystin-LR are reported as effective values, and concentrations of terbuthylazine and cadmium are reported as nominal concentrations

The best descriptive models and the parameters obtained from fitted data are shown in Table 3. Growth rates of *C. vulgaris* after 4 days of exposure to MC-LR and TBA mixtures were fitted to the CA model, obtaining a moderate deviation between observed and CA-predicted responses (SS = 0.43). Including parameter *a* to the CA model to describe synergism or antagonism significantly improved the fit (SS = 0.16, *p*(χ^2^) < 0.001), and revealed antagonism (*a* = 9.08; Tables 1 and 3). Extending the model with parameters *a* and *b*_*DL*_ further reduced the SS value (SS = 0.15, *r*² = 0.86, *p*(χ²) < 0.001) (Table 3). Parameter *a* was positive (*a* = 2.34), suggesting antagonism at low concentrations and synergism at high concentrations, while *b*_*DL*_ was negative (*b*_*DL*_ = -6.57), indicating that the magnitude of antagonism/synergism became effect level dependent, which means that synergism is predicted to occur at concentrations much higher than the ones tested in the experimental design (see Fig. 1; Tables 1 and 3). Therefore, within the tested concentration range, the biological response can be interpreted mainly as antagonistic (Fig. 3A). Similar trends were observed after 7 days of exposure (Table 3; Fig. 3B).

**Table 3:** Summary of the analysis done for the effects on the growth rate of *Chlorella vulgaris* exposed for 4 and 7 days to the binary mixture of microcystin-LR and terbuthylazine, microcystin-LR and cadmium, cylindrospermopsin and terbuthylazine, cylindrospermopsin and cadmium and finally terbuthylazine and cadmium (Chem 1 × Chem 2, respectively)

Mixture	Exposure time (days)	Fit method^a^	Concentration addition^b^							
			Reference		Deviation					
			SS	*r*^2^	Type	*p*(χ^2^)	SS	*r* ^2^	a	b_DR_/b_DR_
Microcystin-LR and terbuthylazine	4	MC-LR drc fix	0.43	0.58	A	1.89 × 10^− 25^	0.16	0.84	9.08	-
					DL	4.59 × 10^− 04^	0.15	0.86	2.34	-6.57
	7	MC-LR drc fix	0.11	0.82	A	6.51 × 10^− 23^	0.05	0.92	3.98	-
					DL	2.34 × 10^− 04^	0.04	0.93	1.25	-2.63
Microcystin-LR and cadmium	4	MC-LR drc fix	0.97	0.57	A	4.08 × 10^− 05^	0.84	0.63	8.59	-
	7	MC-LR drc fix	0.67	0.69	DR	0.03	0.63	0.71	3.21	-7.01
					DL	2.13 × 10^− 06^	0.53	0.75	-4.73	1.23
Cylindrospermopsin and terbuthylazine	4	TBA drc fix	0.40	0.48	A	2.03 × 10^− 23^	0.16	0.80	5.05	-
					DR	1.97 × 10^− 03^	0.14	0.82	2.21	8.19
					DL	6.94 × 10^− 03^	0.15	0.81	0.56	-11.39
	7	TBA drc fix	0.13	0.75	A	7.95 × 10^− 14^	0.08	0.85	2.39	-
					DL	9.46 × 10^− 07^	0.06	0.88	0.02	-120.44
Cylindrospermopsin and cadmium	4	Free	1.45	0.55	A	8.16 × 10^− 09^	1.06	0.67	18.03	-
	7	Free	0.76	0.60	A	2.41 × 10^− 03^	0.70	0.64	13.55	-
Terbuthylazine and cadmium	4	Free	0.23	0.94	A	0.01	0.22	0.94	0.55	-
	7	Free	0.13	0.95	A	0.02	0.12	0.96	0.42	-

^a^The fit method indicates if any parameter had to be fixed during fitting due to the poor single chemical effect data, followed by statistics for the reference model of concentration addition and their best deviation function

^b^The model parameters given are: *a* and *b*_*DR*_*/b*_*DL*_ are the parameters obtained from the deviation functions; SS is the sum of squared residuals; *r*^*2*^ is the coefficient of determination; χ^2^ is the Chi-squared test, and *p*(χ^2^) indicates the outcome of the likelihood ratio test (significance level *p* < 0.05)

^c^A is antagonism; DR is dose ratio deviation and DL is dose level deviation

After 4 days of exposure to MC-LR and Cd, a significant antagonistic effect (SS = 0.84, *r*^2^ = 0.63, *p*(χ^2^) < 0.001, *a* = 8.59) was detected when compared to the CA model (Table 3; Fig. 3C). After 7 days of exposure, a DR-dependent deviation was detected (SS = 0.63, *r*^2^ = 0.71, *p*(χ^2^) < 0.03), with synergism when MC-LR dominated the mixture and antagonism when Cd was the dominant chemical (Tables 1 and 3). However, adding both *a* and *b*_*DL*_ significantly improved the model fit (SS = 0.53, *r*^2^ = 0.75, *p*(χ^2^) < 0.001) and the DL dependent deviation from the CA model was therefore selected as the best-fitting model. The parameter *a* (*a* = -4.73) indicated synergism at low concentrations and antagonism at high concentrations (Fig. 3D). The switch between synergism and antagonism may be calculated from 1/*b*_*DL*_ × EC_50_ (Jonker et al. 2005). Thus, the change occurred at concentrations lower than the EC_50_ level (1/1.23 = 0.81 × EC_50_).

**Fig. 4:**
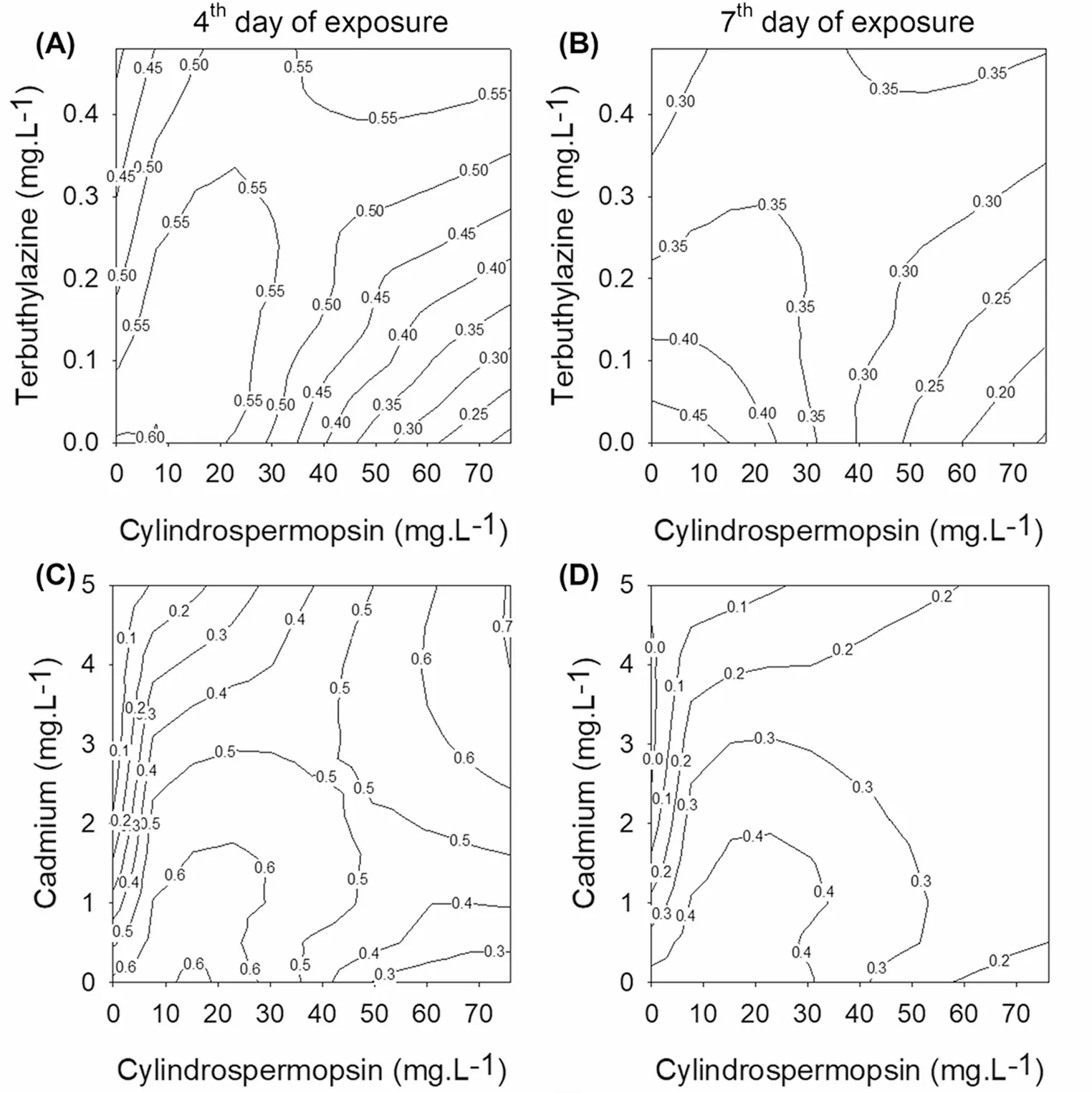
Concentration-response relationship for the binary mixtures of cylindrospermopsin and terbuthylazine and cylindrospermopsin and cadmium tested on the growth rate of *Chlorella vulgaris* after 4 and 7 days of exposure (2D isobolic surface). **A** shows a dose ratio-dependent deviation from the CA model; **B** shows a dose level-dependent deviation from the CA model; **C** and **D** show antagonistic deviations from the CA model fit. Concentrations of cylindrospermopsin are reported as effective values, and concentrations of terbuthylazine and cadmium are reported as nominal concentrations

For the effects of the CYN and TBA mixture on the growth rates of *C. vulgaris* after 4 days, the CA model showed a moderate deviation (SS = 0.40). Adding the parameter *a* significantly improved the fit (SS = 0.16) and showed an antagonistic pattern (*a* = 5.05, *p*(χ^2^) < 0.001) (Tables 1 and 3). A further improvement was obtained after adding the parameters a and *b*_*DR*_, (SS = 0.14, r^2^ = 0.82, p(χ^2^) < 0.05) with a DR-dependent deviation (Table 3). In this case, antagonism was observed and mainly explained when CYN was the dominant chemical in the mixture (*a* = 2.21 and *b*_*DR*_ = 8.19, Tables 1 and 3; Fig. 4A). Although the DL-dependent model was significant, it did not improve the fit compared with the DR-dependent model (Table 3). After 7 days, the CA model showed a good fit to the mixture data of CYN and TBA (SS = 0.13). Adding parameter a improved the fit (SS = 0.08), significantly increased the *r*^2^ to 0.85, and indicated antagonism (*a* = 2.39, *p*(χ^2^) < 0.001, Tables 1 and 3). Incorporating parameters a and bDL further reduced SS (SS = 0.06, *p*(χ^2^) < 0.001), suggesting a DL-dependent deviation, with synergism predicted only at concentrations well above those tested, thus, antagonism dominated the observed response (Fig. 4B).

Regarding the CYN and Cd mixture exposure, CA provided a significant fit (SS = 1.45). Adding parameter *a* led to a small but significant improvement (SS = 1.06, *r*^2^ = 0.67, *p*(χ^2^) < 0.001, Table 3), indicating antagonism (*a* = 18.03) (Fig. 4C). On the 7th day of exposure, data fitted the CA model (SS = 0.76, *r*^2^ = 0.60), but a significantly better fit was obtained after adding parameter *a* (SS = 0.70, *r*^2^ = 0.64, *p*(χ^2^) < 0.05, Table 3), indicating antagonism (*a* = 13.55) (Fig. 4D).

Results on the effects of the TBA and Cd mixture on the growth rate of *C. vulgaris* after 4 days of exposure were well described by the CA model (SS = 0.23, *p* < 0.05).

**Fig. 5:**
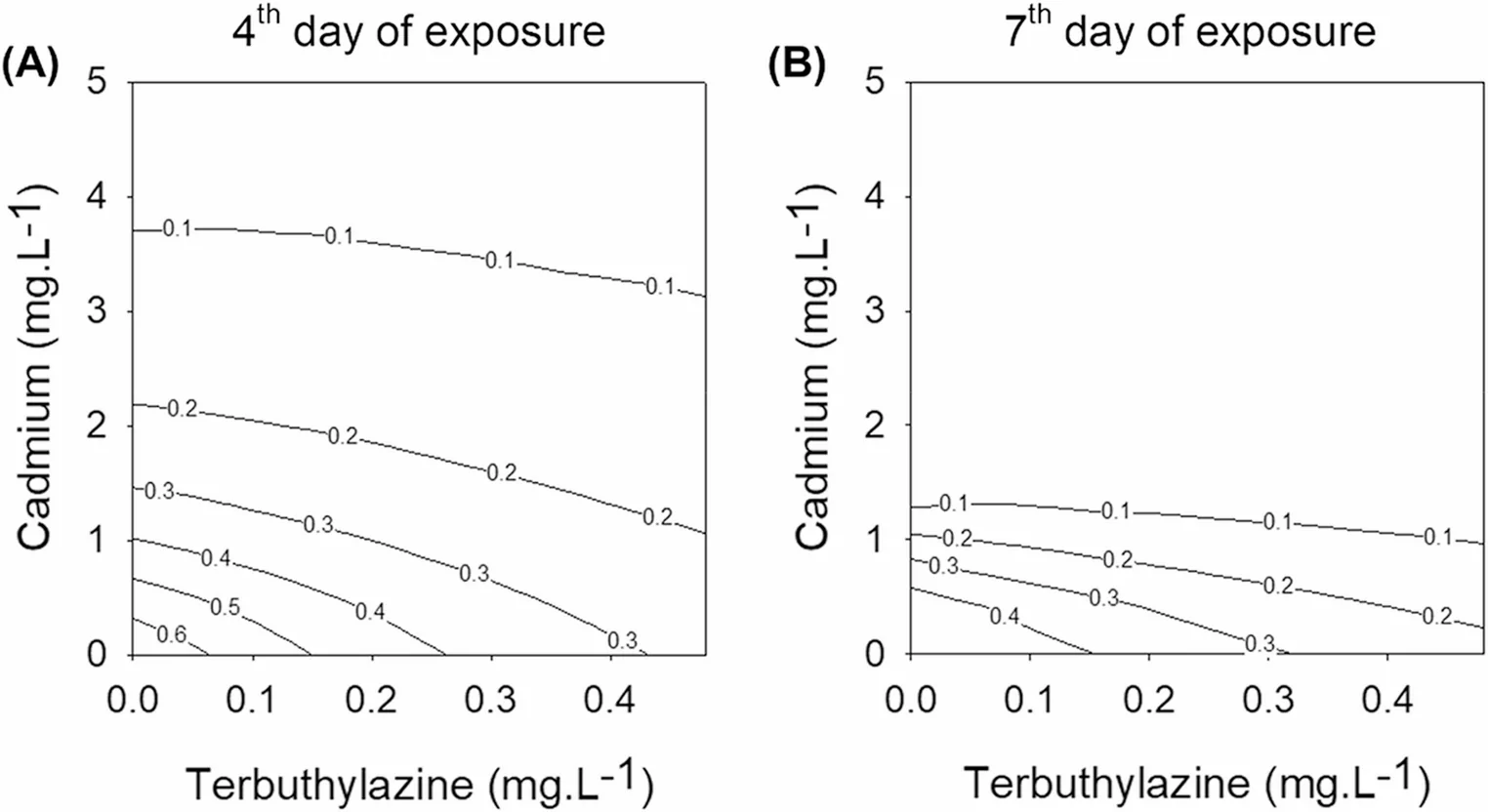
Concentration-response relationship for the binary mixture of terbuthylazine and cadmium tested on the growth rate of *Chlorella vulgaris* after 4 and 7 days of exposure (2D isobolic surface). **A** and **B** exhibit an antagonistic pattern after the CA model is fitted. Concentrations of terbuthylazine and cadmium are reported as nominal concentrations

However, extending the CA model to include the S/A deviation parameter, a slight decrease in the SS value was observed (SS = 0.22, *r*^2^ = 0.94, *a* = 0.55, Table 3) with significant differences at *p*(χ^2^) < 0.05, indicating an antagonistic effect on the growth response(Fig. 5A). After 7 days, a significant antagonistic pattern (SS = 0.12, *r*^2^ = 0.96, *p*(χ^2^) < 0.05, *a* = 0.42) was observed when compared to the CA model (Table 3; Fig. 5B).

As MC-LR did not cause 50% growth inhibition in the single-exposure experiments, EC_50_ values were estimated, and SRs were calculated for each MC-LR concentration tested in the presence of TBA/Cd (Table 4). After 4 days, MC-LR decreased TBA toxicity with SRs of 0.46 at 8.7 mg L^− 1^ and 0.33 at 19.7 mg L^− 1^, but the decreased TBA toxicity was less pronounced after 7 days of exposure. MC-LR also reduced Cd toxicity after 4 days (SR = 0.46 at 19.7 mg L^− 1^), but this effect was less obvious after 7 days (Table 4). While SR describes changes in EC_50_ at specific MC-LR concentrations, MIXTOX evaluates the overall concentration-response curve, revealing a dose-level-dependent interaction, with synergism occurring at low concentrations and significant antagonism at high concentrations (Table 3). In the CYN mixture with TBA, it was not possible to calculate the SRs for either exposure period because TBA did not induce 50% growth inhibition. Although CYN alone induced 50% of growth inhibition in the single exposure experiment (Table 2), the addition of TBA reduced this effect. During the first 4 days of exposure, *C. vulgaris* growth was similar to the control at 0.03–0.06 mg L^− 1^ TBA and was stimulated at concentrations ≥ 0.12 mg L^− 1^. After 7 days, weak growth inhibition (< 30%) was observed at low TBA concentrations (0.03–0.12 mg L^− 1^), whereas growth was similar to the control or stimulated at TBA concentrations of 0.24 mg L^− 1^.

**Table 4:** EC_50_ values (with the standard errors, in mg L^− 1^) and synergistic ratios (SRs) estimated and calculated, respectively, for terbuthylazine (TBA) and cadmium (Cd) when co-occurring with each concentration of microcystin-LR in the mixture experiments

Compound	Exposure time (days)	EC_50_ (± SE)	Microcystin-LR (mg L^− 1^)					
			0	0.4	2.3	5.1	8.7	19.7
TBA	4	SR^a^	0.37 (± 0.03)	0.37 (± 0.03)	0.48 (± 0.05)	0.67 (± 0.10)	0.80 (± 0.17)	1.14 (± 0.48)
		EC_50_ (± SE)	-	1.00	0.77	0.55	0.46	0.32
	7	SR^a^	0.46 (± 0.03)	0.42 (± 0.03)	0.48 (± 0.03)	0.61 (± 0.05)	0.74 (± 0.10)	0.90 (± 0.17)
		EC_50_ (± SE)	-	1.10	0.96	0.75	0.62	0.51
Cd	4	SR^a^	0.92 (± 0.08)	0.75 (± 0.04)	1.76 (± 0.15)	0.96 (n.d.)	0.97 (± 0.25)	2.02 (± 1.13)
		EC_50_ (± SE)	-	1.23	0.52	0.96	0.95	0.46
	7	SR^a^	0.76 (± 0.04)	0.72 (± 0.02)	1.08 (± 0.06)	0.76 (± 0.02)	0.86 (± 0.04)	0.92 (± 0.67)
			-	1.06	0.70	1.00	0.88	0.83

SE is the standard error;

n.d. is not determined;

^a^SR = EC_50 TBA or Cd without MC−LR_ / EC_50 TBA or Cd and MC−LR treatments_

## Discussion

### Single chemical exposures

The single-chemical exposures demonstrated that MC-LR and CYN were toxic to *C. vulgaris* only at high concentrations (Table 2), which are not typically found in aquatic environments (Corbel et al. 2014; Yang et al. 2016; Wang et al. 2021; Du et al. 2022). However, exceptionally high MC concentrations are reported in the literature. For instance, MC concentrations up to 124.46 mg L^− 1^ were measured in water samples in southern Africa, most likely due to increased organic matter from animal faeces combined with seasonal decreases in water levels (Masango et al. 2010).

In the present study, CYN decreased the growth rate of *C. vulgaris* by 50% at 49.21 and 56.34 mg L^− 1^ in a 4-day exposure period, and at 47.67 and 51.26 mg L^− 1^ after 7 days, suggesting a slight increase in toxicity with longer exposure. These results are inconsistent with those of Seifert (2007) and Kinnear et al. (2008, 2010), who reported that CYN concentrations below 100 µg L^− 1^ were considered low risk for aquatic organisms, including green algae. The growth rate of the green microalgae *Parachlorella kessleri* was unaffected by CYN at 55 and 150 µg L^− 1^ (Pereira et al. 2018). Although the toxicity of MC-LR and CYN is likely to occur only at high concentrations, bioaccumulation can occur at low levels of these cyanotoxins, therefore, potential impairments in microalgae growth in long-term exposures should not be disregarded (Kinnear 2010). Overall, LOEC values for MC-LR were 40.4 and 78.9 mg L^− 1^, resulting in maximum growth rate inhibitions of 23% and 44% after 4 and 7 days of exposure, respectively. Stronger effects have been reported for *C. vulgaris* exposed to MC-LR lysates for 96 h, with 35.80% and 55.84% inhibition at 2.5 mg L^− 1^ (LOEC) and 5 mg L^− 1^, respectively (Albuquerque et al. 2022). Babica et al. (2007) also reported lower LOEC values for MC-LR than those reported in this study during the same exposure period (4-day LOEC of 5 mg L^− 1^ and 7-day LOEC of 25 mg L^− 1^ for *Pediastrum duplex* and *Scenedesmus quadricauda*). *Raphidocelis subcapitata* was shown to be the most sensitive microalgae to MC-LR, with LOEC values of 1 mg L^− 1^ for the 4th and 7th days of exposure (Babica et al. 2007).

To our knowledge, this study provides the first evidence that a commercial formulation containing TBA is highly toxic to *C. vulgaris*, reducing its growth rate by 50% at concentrations of 0.35–0.39 mg L^− 1^ in a 4-day exposure period. Longer exposures slightly decreased TBA toxicity with 7-day EC_50_ values of 0.39–0.46 mg L^− 1^, indicating growth recovery. However, the toxicity of TBA, used as a pure compound, to microalgae is already known. Queirós et al. (2018a) reported a 96 h-EC_50_ of 0.177 mg L^− 1^ for the growth inhibition of *C. vulgaris* exposed to TBA, which is half the value obtained in the present study. This difference may be related to methodological differences between the studies, including the test medium (Z8 medium vs. Woods Hole MB medium), initial algal density (5 × 10⁵ cells mL^−1^ vs. 10⁴ cells mL−¹), temperature (25 ± 2 °C vs. 23 ± 1 °C), or the different commercial formulations used. For *R. subcapitata*, the 96 h-EC_50_ value was much lower (0.015 mg L^− 1^). Similarly, Pérez et al. (2011) reported a 3-day EC_50_ value of 0.024 mg L^− 1^ for the growth inhibition of *R. subcapitata*, which is also much lower than the values found in the present study. Okamura et al. (2000) also found a similar 3-day EC_50_ value (0.036 mg L^− 1^) for *R. subcapitata* exposed to TBA. In contrast to previous studies, the commercial TBA formulation used in this study showed less toxicity to microalgae, even though different microalgae species (with varying susceptibilities), exposure periods, and exposure media were employed to assess toxic effects. This differs from reports indicating that commercial formulations are more toxic to aquatic organisms than their active ingredients (Cedergreen and Streibig 2005; Nagy et al. 2020; J. L. Pereira et al. 2000, 2009b).

Cd was highly toxic over time towards *C. vulgaris*, with EC_50_ values ranging from 0.92 to 4.15 mg L^− 1^ after 4 days and 0.76 to 1.80 mg L^− 1^ after 7 days (Table 2). Comparable results are reported in the literature, with a 4-day EC_50_ value of 1.02 mg L^− 1^ (Lam et al. 1999) and an EC_50_ of 0.10 mg L^− 1^ for Cd on *C. vulgaris* (Rachlin and Grosso 1993). Pang et al. (2021) reported EC_50_ values for the growth inhibition of *C. vulgaris* exposed to Cd at concentrations of 2.76 mg L^− 1^ (24 h), 1.08 mg L^− 1^ (48 h), and 0.93 mg L^− 1^ (72 h). Cheng et al. (2016) observed growth inhibition of *C. vulgaris* primarily at Cd concentrations of 3 and 5 mg L^− 1^ for 12 days, with mortality at 7 mg L^− 1^.

### Binary mixture exposures

All the mixture data from the present study deviated from the non-interactive conceptual model CA, suggesting interactions between chemicals rather than additive or independent responses. Such deviations from the CA model may occur when compounds affect one another’s bioavailability, modes of action, or behaviour after uptake (e.g., bioaccumulation) (Loureiro et al. 2010). Although the concentrations used in the mixture exposures were higher than those typically found in the environment, they allow the characterisation of interactions between cyanotoxins and anthropogenic contaminants under controlled laboratory conditions. Subsequently, this approach can be applied to real scenarios by incorporating safety factors to convert non-ecologically relevant chemical levels into more ecologically relevant ones.

Fitting the CA model to *C. vulgaris* growth rate data exposed to a MC-LR and TBA mixture revealed a DL-dependent deviation at both exposure periods, indicating antagonism at the tested concentrations and potential synergism at higher concentrations. The antagonistic pattern is consistent with the SRs, which showed that MC-LR at 5.1–19.7 mg L^− 1^ reduced TBA toxicity by approximately 0.3-0.6-fold. One possible explanation is that MC-LR-induced alterations in *C. vulgaris* may mitigate TBA toxicity through processes associated with photosynthesis. TBA inhibits photosynthetic electron transport in plants and algae by reversibly binding to the D1 protein domain of the PSII reaction centre (Faust et al. 2001; Battaglino et al. 2021). MC-LR at concentrations greater than 10 mg L^− 1^ has also been reported to affect photosynthetic efficiency and energy fluxes within PSII in green algae (Perron et al. 2012). It is also well established that MC-LR induces the production of reactive oxygen species (ROS) and enhances the production of antioxidant enzymes in photosynthetic organisms (Wiegand et al. 2002; Pflugmacher 2004; Mohamed 2008; Bártová et al. 2011; Li et al. 2024). The intracellular increase in ROS may exceed the cellular detoxification capability and lead to the oxidation of biomolecules and preferential damage to PSII, with irreversible oxidation of the D1 protein causing its degradation (Okada et al. 1996; Kim and Lee 2005; Drábková et al. 2007; Krieger-Liszkay et al. 2008). If it occurs, TBA binding to the D1 protein may be reduced, thereby decreasing its inhibitory effect on photosynthetic electron transport.

Antagonism was obtained for *C. vulgaris* growth rate exposed to MC-LR and Cd mixture after 4 days, which may be related to reduced Cd toxicity via the induction of metallothioneins (MTs) and phytochelatins (PCs) (i.e., detoxification proteins in algae) (Perales-Vela et al. 2006) as part of the cellular defence response. The induction of PCs may also be linked to the presence of MC-LR in the mixture since PCs are small metal-binding polypeptides enzymatically synthesised from glutathione (GSH). Wang et al. (2011, 2012) found that combined exposure to MC-LR and linear alkylbenzene sulfonate increased the GSH content. Another possible explanation is the formation of MC-LR-Cd complexes during the 4-day exposure, which could reduce the bioavailability of both compounds to *C. vulgaris*. The formation of MC-metal complexes resulting from the coexistence of metals and cyanotoxins in aquatic systems has been reported (Saito et al. 2008; Klein et al. 2013; Pochodylo et al. 2017). After 7 days, a DL-dependent deviation was observed, indicating synergism at low concentrations and antagonism at high concentrations. Although this pattern was not confirmed by SRs, the DL-dependent deviation was not rejected (*p*(χ^2^) < 0.001). Therefore, the MIXTOX model suggests that this interaction may occur over a broader concentration range than that assessed by the SR approach. One possible explanation for the synergism observed at low concentrations is the combined production of ROS induced by both compounds, which could enhance oxidative stress (Pinto et al. 2003; Mohamed 2008; Bártová et al. 2011) and impair cellular detoxification mechanisms. On the other hand, antagonism at high concentrations may reflect interactions between MC-LR and Cd either in the exposure medium or within the cytoplasmic matrix, or reduced levels of extra detoxification proteins. It is also relevant to highlight the temporal variation in the response pattern of *C. vulgaris* (antagonism observed after 4 days, followed by synergism at low concentrations after 7 days), suggesting that prolonged exposure to MC-LR and Cd may alter the nature of the interaction between both compounds. Cao et al. (2020) reported a synergistic effect of MC-LR in combination with Cd in the macrophyte *Ceratophyllum demersum* L., enhancing Cd bioaccumulation and adversely affecting its growth, total chlorophyll content, and the antioxidative system. Similarly, the interaction between MC-LR and Cu negatively affected antioxidant defence systems and increased bioaccumulation of MC-LR and Cu in the macrophyte *Vallisneria natans*.

Fitting the CA model to *C. vulgaris* data to the CYN and TBA mixture for 4 days revealed a DR-dependent antagonism deviation, particularly when CYN dominated the mixture. One possible explanation for this antagonism is that CYN activates cellular detoxification mechanisms, which may reduce the mixture’s toxicity and contribute to the observed recovery or stimulation of growth. Evidence for the involvement of glutathione-S-transferase (GST) in atrazine metabolism in green algae based on the GSH-dependent formation of water-soluble metabolites has been reported. Therefore, TBA detoxification in *C. vulgaris* may involve similar metabolic pathways (Tang et al. 1998). Studies have reported that GST may be involved in the detoxification of CYN in microalgae (Duval et al. 2005; Chia et al. 2017). Furthermore, CYP450 monooxygenases could also contribute to TBA detoxification in *C. vulgaris*, thereby potentially reducing TBA toxicity in the mixture (Thies et al. 1996).

After 7 days of exposure, a DL-dependent deviation was obtained, indicating antagonism at low concentrations and synergism at high concentrations. According to the MIXTOX tool output, the shift from antagonism to synergism would occur only at CYN and TBA concentrations higher than those tested in the present study. Therefore, within the tested concentration range, the biological response can be interpreted predominantly as antagonistic (Runnegar et al. 1995), which may be associated with the detoxification processes proposed above.

For the CYN and Cd mixture, an antagonistic deviation from the CA model was also observed after 4 and 7 days of exposure, which may be related to the induction of MTs and PCs by Cd and to CYN detoxification via GST. Potential interactions between CYN and Cd in the exposure medium or within the cytoplasmic matrix, similar to those proposed for MC-LR and Cd mixture, cannot be excluded (Terao et al. 1994; Shaw et al. 2000; Conner and Schmid 2003; Pinto et al. 2003; Leonard et al. 2004; Valko et al. 2005; Badisa et al. 2007; Froscio et al. 2001, 2008; Beyer et al. 2009). To our knowledge, only the study by Casas-Rodríguez et al. (2024) has evaluated the cytotoxicity of the binary combination of CYN and Cd, using human immune cell lines, reporting antagonism at all concentrations in Jurkat and THP-1 cells, with a slight shift towards additivity at 24 h and 48 h (Casas-Rodríguez et al. 2024).

TBA and Cd mixture also showed antagonism at 4 and 7 days, which may be related to reduced toxicity of both compounds through the induction of MTs and PCs in the case of Cd, and of GSH, GST, and possibly CYP450 monooxygenases in the case of TBA. Antagonism was also detected in the mixture of TBA and the chloroacetanilide herbicide metolachlor on the growth rate of the green algae *R. subcapitata* (Faller et al. 2005; Pérez et al. 2011), while no deviation from the additive conceptual models was observed for the growth rate of *R. subcapitata* exposed to a combination of TBA and chlorpyrifos (Silva et al. 2018).

To summarise, antagonism was the dominant deviation from the CA model after 4 days across all tested mixtures. After 7 days, the DL-dependent deviation prevailed. In the binary mixtures of MC-LR/TBA and CYN/TBA, a decrease in mixture toxicity (antagonism) was observed at the lower concentrations tested (environmentally relevant concentrations). The opposite trend was observed in the MC-LR/Cd mixture. To our knowledge, our study provides the first report of antagonistic interactions between CYN and TBA/Cd affecting the growth of *C. vulgaris*. Information about the combined modes of action of TBA and Cd is scarce, even though the combined toxicity of TBA or Cd and other anthropogenic pollutants has been investigated (Rachlin and Grosso 1993; Lam et al. 1999; Munkegaard et al. 2008; Pérez et al. 2011; Queirós et al. 2018b; Silva et al. 2018; Luo et al. 2019; Miranda et al. 2019; Hadjadji et al. 2025).

The antagonism observed at shorter exposure periods (4 days) suggests that the simultaneous presence of cyanotoxins and anthropogenic compounds in aquatic systems may result in lower-than-expected toxicity compared with single-chemical exposures under the tested conditions, and may therefore have the potential to result in less severe ecological effects. On the other hand, the synergism obtained in this study at low concentrations in longer exposure periods (7 days) (i.e., MC-LR and Cd mixture) suggests that the simultaneous presence of cyanotoxins and anthropogenic pollutants in the aquatic environment may lead to more toxic effects than expected from the single-chemical exposures, which could potentially translate into more severe ecological effects. Nevertheless, these interpretations are based on laboratory experiments using concentrations that exceed those typically found in the environment. Therefore, further research is needed to test the proposed mechanisms underlying these interactions and to determine their ecological relevance under environmentally realistic scenarios.

## Conclusion

This study investigated the toxicity of single-chemical exposures and binary mixtures to the growth of *C. vulgaris*. The results revealed that TBA was the most toxic compound, followed by Cd, CYN, and MC-LR. Results for the binary mixtures show that antagonism was the predominant interaction after 4 days of exposure, whereas more complex dose-level and dose-ratio-dependent interactions were observed after 7 days, including a time-dependent shift from antagonism to synergism (at low concentrations) for the MC-LR and Cd mixture between 4 and 7 days of exposure. Given the expected increase in the frequency and magnitude of HABs and co-exposure to cyanotoxins and anthropogenic contaminants, this time-dependent change in toxicity patterns may represent a concern for aquatic organisms in contaminated habitats. The MIXTOX tool effectively modelled these interactions, identifying dose-level and dose-ratio-dependent deviations from the CA model. These findings stress the need to account for mixture effects in ecotoxicological assessments, as combined toxicities can differ significantly from their individual effects on algal growth. Further studies are needed to elucidate the mechanisms underlying these binary mixture interactions and to improve predictions of interactions between cyanotoxins and anthropogenic contaminants in aquatic organisms under environmentally relevant exposure conditions.

## Data Availability

All data supporting the findings of this study are available in the Zenodo data repository under the identifier https://doi.org/10.5281/zenodo.15395705.
